# Patterns of protist diversity associated with raw sewage in New York City

**DOI:** 10.1038/s41396-019-0467-z

**Published:** 2019-07-09

**Authors:** Julia M. Maritz, Theresa A. Ten Eyck, S. Elizabeth Alter, Jane M. Carlton

**Affiliations:** 1 Department of Biology, Center for Genomics and Systems Biology, New York University, 12 Waverly Place, New York, NY 10003 USA; 2 Department of Biology, York College, City University of New York, New York, NY USA; 3 The Graduate Center of the City University of New York, 365 Fifth Avenue, New York, NY 10016 USA

**Keywords:** Microbial ecology, Microbial ecology

## Abstract

Protists are ubiquitous components of terrestrial and aquatic environments, as well as animal and human microbiomes. Despite this, little is known about protists in urban environments. The ~7400-mile sewer system of New York City (NYC) collects human waste from ~8 million human inhabitants as well as from animals, street runoff, and groundwater, providing an ideal system to study these microbes. We used 18S rRNA amplicon sequencing and shotgun metagenomic sequencing to profile raw sewage microbial communities. Raw sewage samples were collected over a 12-month period from 14 treatment plants of the five NYC boroughs, and compared with samples from other environments including soil, stormwater, and sediment. Sewage contained a diverse protist community dominated by free-living clades, and communities were highly differentiated across environments. Seasonal differences in protist composition were observed; however, network analysis and functional profiling demonstrated that sewage communities were robust and functionally consistent. Protists typically associated with human and animal guts or feces were frequently detected. Abundance of these parasites varied significantly both spatially and temporally, suggesting that spikes could reflect trends in the source population. This underscores sewage as a valuable model system for monitoring patterns in urban microbes and provides a baseline protist metagenome of NYC.

## Introduction

Studies investigating microbial diversity (the “urban microbiome”) in urban air [1], rodents [2], urban soils [3–5], urban waters [6], surfaces within urban transit systems [7, 8], and ATM keypads [9] are increasingly common but a wide range of urban landscapes remain to be investigated. In particular sewage systems that collect and transport human waste from residential, commercial, and industrial toilets to wastewater treatment plants are integral to urban infrastructure while also serving as a reflection of urban ecology, but have been little studied. Some recent studies have demonstrated that the influent of these systems serves as a proxy of population-level fecal microbiota within a city, retaining microbial signatures of human inhabitants including both commensal taxa and pathogens [10–13]. Systems where the sewer and storm drains are combined accumulate further microbes from animal wastes and natural environments through groundwater and surface runoffs [6]. Microbial communities of combined sewage thus consist of composite populations, whose presence and relative abundance should reflect the surrounding environment. Urban sewage infrastructure also contains its own unique nonfecal microbial communities that are rarely found in influent communities [14], forming identifiable communities that demonstrate significant geographical and seasonal trends, and serve as alternate indicators of sewage discharge into the environment [15].

Protists are important components of trophic chains and nutrient cycles in terrestrial and aquatic environments as well as members of human and animal microbiomes, where their relationships with their hosts vary from parasitic to mutualistic [16–18]. Protists can also serve as indicators of water quality, contaminant levels, and habitat alterations in both built and natural environments [19]. This includes human-made ecosystems such as wastewater treatment facilities, where they play roles in the purification process [20]. As protists are critical in many environmental processes, these communities potentially have important ecological roles in cities as well as potential public health consequences, but broad surveys of protists in urban environments are scarce. The diversity of protists in sewage has been explored on a very limited basis, and their composition has unsurprisingly been shown to reflect contributions from different animal, human, and environmental sources [21].

New York City (NYC) is the most populous city in US, whose more than eight million people occupy ~300 sq. miles divided amongst five boroughs. This unique urban landscape features the highest population density of any major city in US; distinct populations of human-associated vertebrate and invertebrate commensals; intensive land use for recreational and industrial purposes; and a ~7400-mile combined sewer system, maintained and operated by the NYC Department of Environmental Protection (DEP), that handles ~1.3 billion gallons of wastewater daily. We believed that high-throughput sequencing of sewage from the NYC combined sewage system would characterize the diversity of protists in New Yorkers as well as provide a baseline of protists found in the environment of NYC, highlighting the utility of sewage as a valuable model system for monitoring urban microbes. Here, we present the first city-scale inventory of protists in untreated sewage in the five boroughs of NYC using a combination of 18S rRNA gene amplicon and shotgun metagenomic sequencing. We also compare the protist community composition of sewage to that of other NYC environments, including soil from parks and green spaces, stormwater, and sediment.

## Materials and methods

### Sample collection, processing, and DNA extraction

**Fig. 1:**
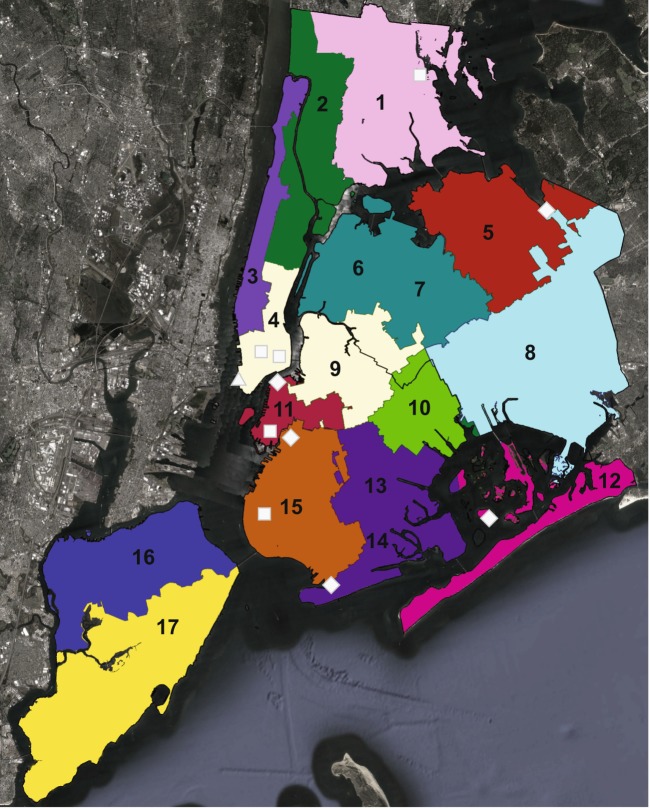
Geographical location of samples. Colors represent DEP drainage areas, and each number represents one of the 17 raw sewage samples. Black lines show NYC borough boundaries; white shapes indicate locations of samples from other environments; triangle = private building; square = soil; diamond = sediment. DEP drainage areas were adapted from the official NYC DEP plant map: http://www.nyc.gov/html/dep/html/wastewater/wwsystem-plantlocations_wide.shtml

Seventeen 250 mL samples of raw sewage from all 14 NYC DEP wastewater treatment plants were collected at four time points over one year (November 2014 and February, May, and August 2015) for a total of 68 samples (Fig. 1; two samples representing smaller drainage areas were collected from three of the 14 plants, i.e., 6 and 7, 4 and 9, and 13 and 14 in Fig. 1). Each sample was collected as part of the DEP’s routine monitoring and represents a composite daily sample, i.e., a combination of raw sewage taken every 3 h over a 24 h period. Samples were refrigerated for up to 24 h prior to pickup, and ~1 mL of raw sewage used for DNA extraction. Two 50 mL samples of stormwater were collected from the roof tank of a private apartment building in lower Manhattan in July 2014, and ~10 mL used for DNA extraction (Fig. 1). Composite (1–5 L total) soil samples were collected from seven sites (four community gardens and three publicly accessible plots, Fig. 1) in October and November 2014, and ~0.5 g of soil from each sample used for DNA extraction. One liter of sediment was collected from the Gowanus Canal in Brooklyn in December 2014, and ~1 mL was used for DNA extraction. Six 10–15 mL surface sediment samples representing two salt marshes and two mudflats were collected in October and November 2014 (Fig. 1). Additional details regarding sample collection and processing are described in detail in Supplemental Methods. Metadata were collected for each sample describing the environment and collection site type, borough, date, and biomass (DNA concentration in ng/uL) (Table S1). Chemical and physical parameters (conductivity, chloride levels, carbonaceous biochemical oxygen demand, and total suspended solids), and plant-specific data regarding the average flow, drainage area size, and population served based on the 2010 census were also obtained for the DEP sewage samples.

DNA extractions were performed in a laminar flow hood on the day of sample pickup using the PowerSoil DNA Isolation kit (QIAGEN, catalog #12888). Two extraction control samples, using PCR-grade water as input material, were performed for each kit lot used. All samples were homogenized prior to processing and two biological replicates were generated per sample.

### 18S rRNA gene amplification and Illumina sequencing

To better capture diversity, both V4 and V9 variable regions of the 18S rRNA gene (referred henceforth as V4 probe and V9 probe) were amplified from all 168 DNA extractions following the Earth Microbiome protocol [22] and the protocol outlined in our previous work [23] and described in detail in Supplemental Methods. Sequencing was performed as described in [23].

### Shotgun metagenomic Illumina sequencing

DNA from raw sewage samples collected in November 2014 was also used to generate shotgun metagenomic libraries. Libraries were constructed as described in Supplemental Methods. Sequencing was performed on two lanes of a HiSeq Rapid Run with 2 × 250 bp paired-end chemistry, resulting in an average sequencing depth of 21.5 × 10^6^ ± 4.2 × 10^6^ paired-end reads per sample.

### Filtering and processing of Illumina 18S rRNA gene amplicon sequences

Raw Illumina reads were trimmed off adapter sequences using the palindrome setting of Trimmomatic (v0.36) [24]. For the V9 data, surviving paired-end reads were joined using fastq-join (v1.1.2) [25] within QIIME (v1.9.0) [26] with a minimum overlap of 2 bp and allowing a 20% error rate in the overlapping area. Preprocessing of V4 Illumina reads was undertaken as outlined in [23]. We also included the sequence data from sewage from a private apartment building in Manhattan described in [23] in our analysis. Demultiplexed reads for both regions were subject to de novo chimera checking, removal of singletons, and clustered into de novo OTUs at 98% identity with UPARSE (USEARCHv8.0.1) [27] as discussed in [23]. Taxonomy was assigned to representative sequences in two steps, as outlined in [23]. The resulting OTUs were filtered to exclude bacterial, archaeal, metazoan, streptophyte, and multicellular fungi sequences based on taxonomic assignments. Datasets for both regions were subsequently filtered to remove low abundance OTUs making up <0.001% of reads in the total dataset [28]. This resulted in 62,054,757 high-quality sequences and 3781 unique OTUs in the V4 dataset and 55,638,370 sequences and 3471 OTUs in the V9 dataset.

### 18S rRNA gene amplicon sequence data analysis

Alpha and beta diversity analyses and statistical comparisons were conducted with QIIME and R packages vegan (v2.4-3) [29] and Phyloseq (v1.20) [30] and described in detail in Supplemental Methods. All other downstream analyses were performed on sum-normalized OTU tables. Tests for differentially abundant taxa with respect to environment were performed using LEfSe [31] and GraPhlAn [32] was used for visualization of results. Communicable disease data were obtained from the New York State Department of Health Communicable Disease Annual Reports (https://www.health.ny.gov/statistics/diseases/communicable/).

Network analysis was performed in R using SpiecEasi (v0.1.2) [33] on per season OTU tables filtered to remove OTUs present in less than one third (eleven) samples. Topological properties of the networks were analyzed in R with iGraph (v1.1.2) [34] and described in detail in Supplemental Methods. The importance of individual OTUs (nodes) within each of the eight networks was determined by plotting the degree versus closeness centrality for each node, and nodes that had the highest values in each network for both metrics were considered potential keystone species.

### Functional profiling of metagenomes

Functional profiles were inferred from raw sequencing data using the whole metagenome shotgun processing workflow from bioBakery workflows (v0.3.3) [35] using default parameters with kneaddata (v0.6.1), MetaPhlAn2 (v2.6.0) [36], and HUMAnN2 (v0.11.1) [37]. After removal of reads that mapped to human and ribosomal rRNA genes, 19–73% (mean 55%) of the remaining reads per sample were mapped to 898,444 UniRef90 [38] gene families, which were collapsed into 459 MetaCyc pathways [39]. UniRef90 proteins that did not map to any MetaCyc pathways were not analyzed further. MetaCyc pathway abundances, in reads per kilobase, were sum-normalized prior to visualization. Heatmaps were created with Hclust2 using average linkage clustering of the Euclidean distance for samples and pairwise Spearman correlation between pathways. Abundances were log_10_ transformed prior to clustering.

## Results

### Cross-sectional sampling of protist communities reveals high differentiation between environments

**Fig. 2:**
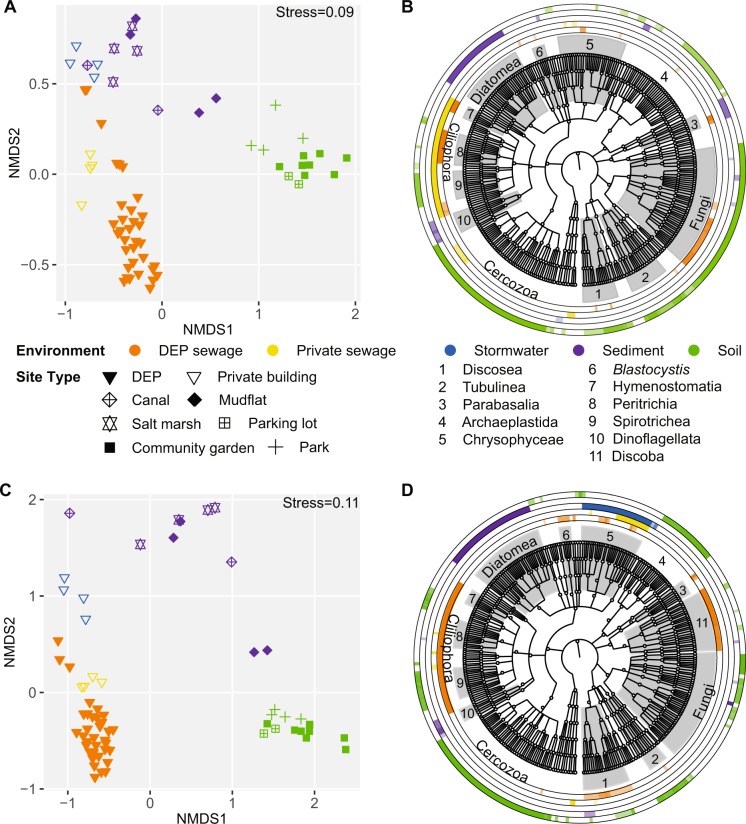
Beta diversity and LEfSe enrichment of protist taxa in five different environments. **a**, **b** V4 region. **c**, **d** V9 region. Beta diversity ordinations (**a**, **c**) were calculated from NMDS of the Bray–Curtis dissimilarity. Cladograms (**b**, **d**) show significant associations of protist taxa with an environment; each of the five environments is represented by a different ring. In all panels colors represent the environment from which each sample was collected, and shape represents the collection site; shading is proportional to the LDA score

We undertook 18S rRNA amplicon sequencing of the V4 and V9 regions of 17 raw sewage samples collected from 14 DEP wastewater treatment plants from the five NYC boroughs in November 2014, and compared them with an additional 16 NYC samples including raw sewage and stormwater from a private apartment building in Manhattan, soil from community gardens and parks in Manhattan, and sediment samples from several estuaries and the Gowanus Canal in Brooklyn (Fig. 1). First, we compared community compositions to determine if DEP sewage differed from the other NYC environments. Collection environment proved to be the major driver of protist diversity and community structure. Biomass yields were highest from soil samples (20.65 ± 21.28 ng/uL) and lowest from DEP sewage samples (0.366 ± 0.349 ng/uL) (Fig. S1A). Alpha diversity of soil samples was significantly higher than that of all other environments (*p* < 0.005 V4 and *p* < 0.05 V9, Wilcoxon rank sum test after FDR correction; Fig. S1B, C), but was only moderately correlated with biomass (Spearman’s rho = 0.36 V4 and 0.45 V9). Beta diversity analysis revealed distinct clustering patterns between environments, and within each environment samples clustered based on collection site (Fig. 2a, c). For both the V4 and V9 data, environment was the major explanatory variable of protist microbiota (adonis *R*^2^ = 0.42 V4 and 0.48 V9, *p* < 0.0005). The borough (geographical location) was only a minor factor in protist community structure across environments (adonis *R*^2^ = 0.09 V4 and 0.08 V9, *p* < 0.0005), and between DEP sewage samples once the environment variable was taken into account, since stormwater, soil, and sediment samples were not obtained from all boroughs (Fig. S2A, B).

LEfSe univariate analyses revealed that while taxa from similar higher-level clades were found across environments, different clades dominated (Table S2). For example, diatomea and dinoflagellata were enriched only in sediment samples (Fig. 2b, d), while cercozoans, tubulinea (amoebozoa), and photosynthetic protists (archaeplastidia) were almost exclusively enriched in soil samples, with some overlap with sewage samples. Members of other clades, such as the chrysophytes, ciliates, fungi, and the discobids (excavata, V9 data only, Fig. 2d) were enriched across multiple environments. DEP sewage samples were notable for the presence of human and animal associated taxa including, *Blastocystis* and parabasalids.

### Sewage protist communities are dominated by free-living taxa

**Fig. 3:**
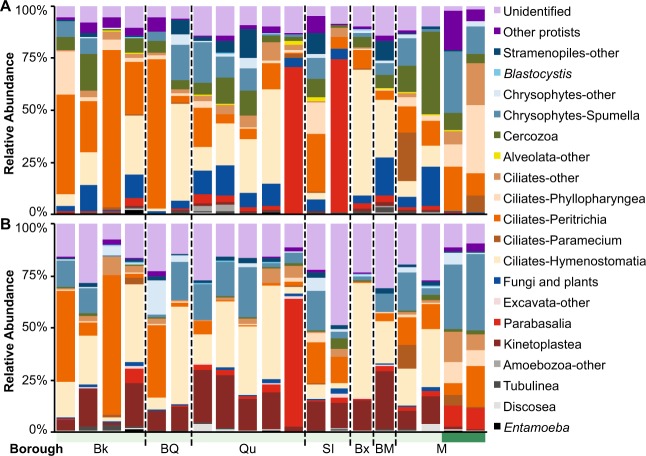
Relative abundance of protist groups identified in DEP and private sewage collected in November 2014. **a** V4 region. **b** V9 region. In both panels data are the average of two replicate samples. Colored horizontal bars indicate DEP (light blue) and private sewage (dark blue) samples. Dotted lines separate samples by borough. Abbreviations: Bk = Brooklyn, BQ = Brooklyn/Queens, Qu = Queens, SI = Staten Island, Bx = Bronx, BM = Bronx/Manhattan, M = Manhattan

Next, we calculated the relative sequence abundance of taxonomic assignments for all sewage samples and both 18S rRNA gene variable regions. NYC sewage contained a diverse protist community dominated by free-living clades (Fig. 3) including ciliates, chrysophytes, cercozoans, and kinetoplastids. The samples were dominated by oligohymenophorean ciliates, particularly members of peritrichia and hymenostomatia, each of which comprised up to 75% of the protist community. The taxon with the highest mean relative abundance (12.5% V4 and 15.4% V9) detected by both variable probes was *Dexiostoma campyla* (hymenostomatia), a free-swimming bacterivorous ciliate found in extremely contaminated freshwater environments, including sewage treatment plants [20]. Other ciliate taxa with high relative abundances (1–4% both regions) include species of *Tetrahymena* and *Vorticella*, common inhabitants of sewage [20, 40]. Chrysophytes, primarily *Spumella-*like flagellates that are widely distributed bactivorous protists [17, 41], also had high relative abundances (mean 6.5% V4, mean 9.5% V9). The V4 probe detected high relative abundances of common soil protists, including cercozoans (mean 8.7%) and fungi (mean 7%) (Fig. 3a) in most DEP samples, while the V9 probe identified increased relative abundances of kinetoplastids (mean 14.3%), primarily the flagellates *Bodo* and *Parabodo* (Fig. 3b) [17, 42, 43], a difference that is likely due to known primer bias between the probes [23, 44]. In contrast, private sewage was dominated by chrysophytes (*Spumella-*like flagellates) that make up 15–37% of the protist community. Private sewage also had a different ciliate profile compared to DEP sewage, with higher relative abundances of the phyllopharyngea and much lower relative abundances of hymenostomatia.

We detected several human- and animal-associated taxa in sewage (Fig. 3). Species of *Cryptosporidium*, an apicomplexan intestinal parasite of vertebrates [45], were present at very low relative abundances (<0.1%) in a small number of samples, and *Toxoplasma*, a ubiquitous zoonotic parasite of mammals and birds [46], was only detected in private sewage. Others, such as species of *Entamoeba* and *Blastocystis*, among the most common intestinal protists of humans and animals [18], were frequently present at low relative abundances (<1%), but were detected at higher relative abundances in some samples from Brooklyn and Queens. Several parabasalids (excavata) including *Tritrichomonas feotus*, a zoonotic parasite found in cattle, domestic cats, and immuno-compromised humans [47–49], *Dientamoeba fragilis*, a common human intestinal parasite [50], and a nondescript *Trichomonas* species closely related to the human parasite *Trichomonas vaginalis* and that was originally isolated from avian sources [51], were also detected in sewage. Parabasalid abundance varied greatly across samples. For example, both variable regions detected a very high relative abundance (>50%) of parabasalids in one sample from Queens, which at the OTU level is represented by *Trichomonas sp*. (~30–40%) and *Tritrichomonas foetus* (~20–30%). The V4 data (but not V9 data) recovered a similar trend in an additional sample from Staten Island (Fig. 3a).

### Functional profiles of sewage are more consistent than taxonomic profiles

**Fig. 4:**
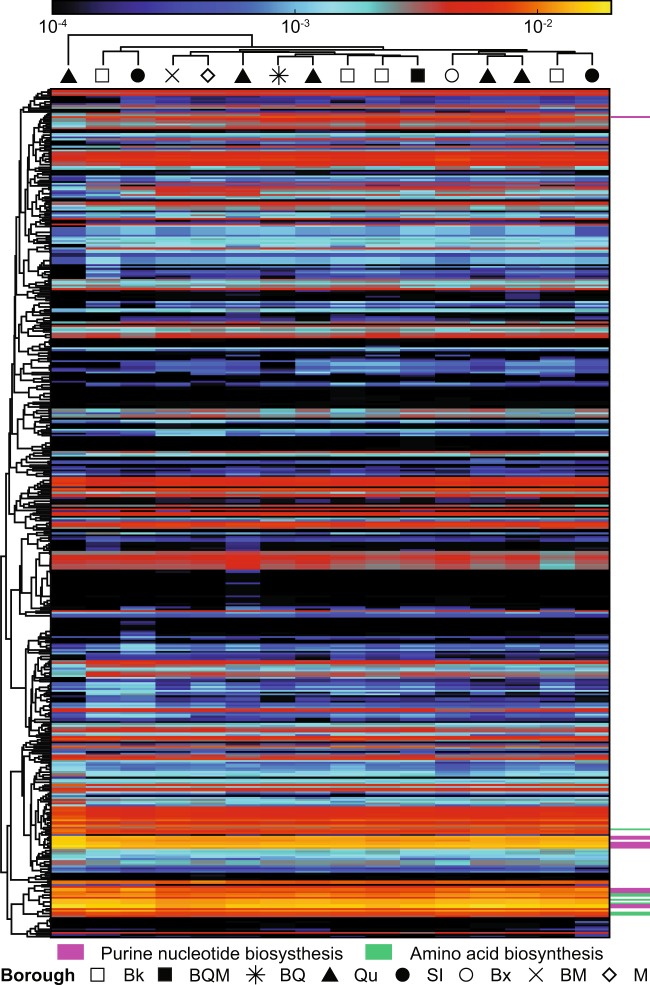
MetaCyc pathways inferred from shotgun metagenomic sequence data in November 2014 DEP sewage samples. Rows represent MetaCyc pathways identified with HUMAnN2, columns represent samples, and cells are colored according to the number of sum-normalized reads per kilobase on a log scale. Members of the most abundant pathway classes are indicated by colored bars to the right of the heat map. Shapes represent the borough from which each DEP sample was collected. Abbreviations: Bk = Brooklyn, BQM = Brooklyn/Queens/Manhattan, BQ = Brooklyn/Queens, Qu = Queens, SI = Staten Island, Bx = Bronx, BM = Bronx/Manhattan, M = Manhattan

We used HUMAnN2 to infer functional profiles from shotgun metagenomic sequence data from 16 of the 17 DEP raw sewage samples collected in November 2014. A total of 898,444 UniRef90 gene families were quantified, which collapsed into 459 MetaCyc pathways. Most of the sewage samples were functionally similar regardless of geography (Fig. 4). The most abundant pathways in sewage were purine nucleotide biosynthesis (Fig. 4, purple) and amino acid biosynthesis (Fig. 4, green). Certain of these pathways, e.g., adenosine ribonucleotides de novo biosynthesis (PWY-7219), guanosine deoxyribonucleotides de novo biosynthesis (PWY-7222), L-isoleucine biosynthesis I (from threonine) (ILEUSYN-PWY), and L-valine biosynthesis (VALSYN-PWY) were present in all samples at levels > 1%. Other highly abundant pathways include pyruvate fermentation to isobutanol (engineered) (PWY-7111), queuosine biosynthesis (PWY-6700), and aerobic respiration (cytochrome c) (PWY-3781). A total of 286 pathways were present in all 16 sewage samples and made up >97% of functional composition of each sample. Hierarchical clustering of sewage samples showed no borough-based patterns in composition (Fig. 4). One sample from Queens, however, clustered separately from the rest—the same sample that showed a very high abundance of parabasalids in the taxonomic data from both 18S rRNA gene variable regions.

### Time-series sampling of protist communities in NYC sewage shows seasonal differences in composition

We collected 17 raw sewage samples from the same 14 DEP sewage treatment plants at three months intervals: winter (February), spring (May), and summer (August) and combined the data with the fall (November) data. Biomass yields were significantly lower for fall samples (0.37 ± 0.35 ng/uL) than those from other seasons (*p* < 0.001, Wilcoxon rank sum test after FDR correction), and highest for winter samples (2.99 ± 3.60 ng/uL) (Fig. S3A). These values were not significantly correlated with total suspended solids measured by the DEP (Pearson’s *r* = 0.15), or the alpha diversity of either variable probe (Table S3). The alpha diversity of summer sewage samples was higher than most other seasons, though this difference was not statistically significant (Fig. S3B, C). No strong correlations were observed between alpha diversity estimates and any of the environmental parameters (conductivity, chloride, carbonaceous biochemical oxygen demand, and total suspended solids) measured by the DEP or plant-specific data (average flow, area, and population served) (Table S2). Significant correlation was however found between the alpha diversity estimates of both variable probes (Spearman’s rho = 0.41, *p* < 0.001).

**Fig. 5:**
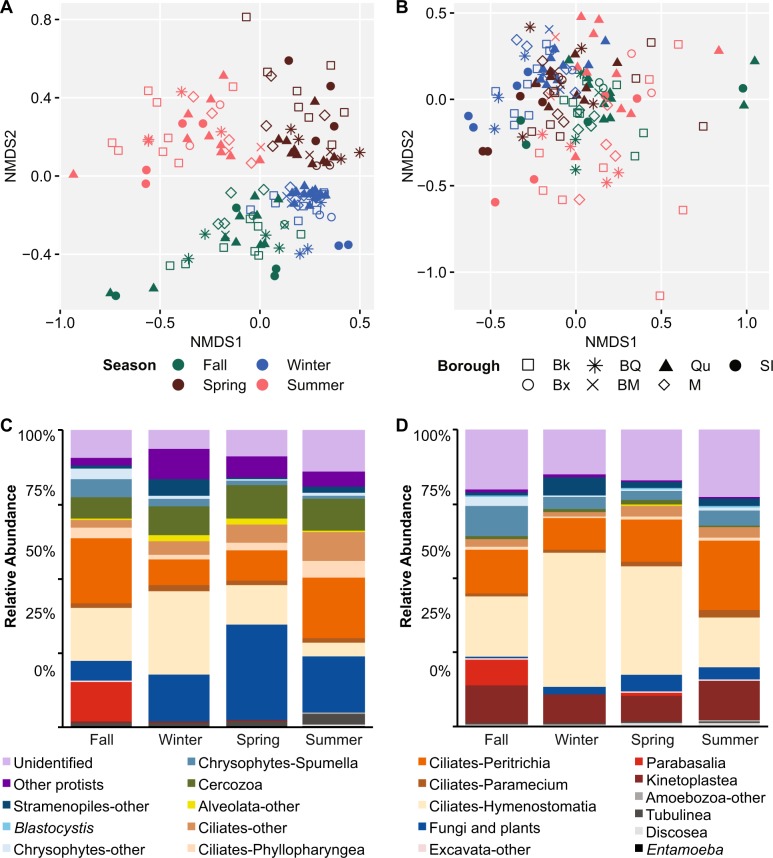
Diversity of DEP sewage over time. Both beta diversity ordinations are from NMDS of the Bray–Curtis dissimilarity for all 136 DEP samples by season and borough. **a** V4 region. **b** V9 region. In both panels colors indicate collection season and shapes represent the borough from which each sample was collected. Abbreviations: Bk = Brooklyn, BQ = Brooklyn/Queens, Qu = Queens, SI = Staten Island, Bx = Bronx, BM = Bronx/Manhattan, M = Manhattan. Relative abundance of protist groups identified in DEP sewage over four seasons from 18S rRNA amplicon sequencing. **c** V4 region. **d** V9 region. In both panels data are the average of all 34 samples collected per season

Beta diversity analysis revealed a clear seasonal pattern across sewage samples (Fig. 5a, b, adonis *R*^2^ = 0.20 V4 and 0.13 V9, *p* < 0.0005). This pattern was particularly evident in the V4 data (Fig. 5a). Communities showed high variability among samples from the fall, spring, and summer, while samples from the winter were more constrained, which is comparable with seasonal results from studies of planktonic microbial eukaryotes [52]. No obvious clustering was observed based on borough, though adonis tests revealed these sample groupings to be significant (*R*^2^ = 0.09 V4 and 0.11 V9, *p* < 0.001). This suggests that there may be differences in the sewage microbial community that are not revealed in broader community comparisons such as nonmetric multidimensional scaling (NMDS).

Analysis of the core sewage community across all 14 plants and four seasons showed that 84 out of 3673 OTUs and 13 out of 2368 OTUs, representing 34.4% and 31.9% of the total reads recovered from sewage, were found in all 136 sewage samples for the V4 and V9 probes, respectively (data not shown). If OTUs present in ≥95% of the samples were included, this increased to 119 and 77 OTUs representing 47% and 59.3% of the total reads for the V4 and V9 regions, respectively. The majority of reads from these core OTUs represent taxa such as ciliates (*Dexiostoma campyla*, peritrichia), chrysophytes, cercozoans, and kinetoplastids that remained dominant throughout the year (Fig. 5c, d). Other core OTUs, which did not have high relative abundances, correspond to human-associated taxa including *Entamoeba* and *Blastocystis* (V9 data only) and free-living amoebozoa (*Vermamoeba vermiformis*), which are widespread in natural and built environments and common hosts of human pathogens [53, 54]. A total of 83 of the core OTUs in the V4 data (4.1% of total reads) and 47 OTUs in the V9 data (6.9% of total reads) could not be identified taxonomically. A similar comparison showed that 2.9–92.2% of the total reads per sample in the V4 data and 5.3–92.9% (mean 62.4%) in the V9 data were from core OTUs present in ≥95% of samples.

### Sewage protist networks are robust

For each season we performed a network analysis using SpiecEasi, and calculated graph topology statistics to assess the overall similarity and trends of the networks as a function of collection season and data set. The V4 networks included larger numbers of OTUs than the V9 networks, but all eight networks were unfragmented with short average path lengths, small diameters, and low levels of connectivity and centralization (Table 1). Low values are consistent with short distances between nodes, close proximity of nodes to each other, and a relatively uniform distribution of these properties between all nodes within the network, which suggests that the networks can be robust to disturbances [55]. The similarity of both whole network properties (number of clusters, mean distance, diameter, and centralization) and average node properties (mean degree, density, and mean closeness centrality) regardless of variable region or collection season suggests that while individual interactions may change, the overall sewage network remains robust.

**Table 1:** Network topology measures of per season networks for the V4 and V9 regions

	V4				V9			
	Fall	Winter	Spring	Summer	Fall	Winter	Spring	Summer
No. nodes	1131	1123	1033	950	773	931	925	816
No. edges	13,468	12,819	9750	8589	6808	8651	9111	7470
No. clusters	1	1	1	1	1	1	1	1
Mean distance	2.71	2.70	2.84	2.80	2.75	2.78	2.73	2.74
Diameter	5	6	6	6	5	5	5	5
Mean degree	23.82	22.83	18.88	18.08	17.61	18.58	19.70	18.31
Max degree	48	42	34	32	30	32	33	32
Degree centralization	0.021	0.017	0.015	0.015	0.016	0.014	0.014	0.017
Density	0.021	0.020	0.018	0.019	0.023	0.020	0.021	0.022
Mean CC	0.373	0.372	0.356	0.359	0.365	0.361	0.368	0.367
Closeness centralization	0.110	0.090	0.077	0.074	0.085	0.076	0.083	0.077

Nodes correspond to OTUs and edges to predicted interactions between OTUs

*CC*  closeness centrality

We also identified OTUs that may play central roles in the sewage microbial networks, commonly referred to as “keystone species.” For each network we identified nodes with a high degree (referred to as network “hubs”), as well as nodes with a high closeness centrality (which can rapidly affect other nodes due to their proximity) [56]. These metrics were identified because they illustrate both the number of connections and how important those connections are to the overall network. The top OTUs by degree were not always the top OTUs by closeness centrality, and few potential keystone taxa were identified (Fig. S4). The OTUs selected as keystone species are presented in Table 2. These OTUs belonged to a variety of free-living protist clades and some unidentified OTUs and were not the same within or between variable regions or seasons.

**Table 2:** Degree, closeness centrality (CC), and taxonomy of OTUs selected as keystone species for per season networks for the V4 and V9 regions

Region	Season	Degree	CC	Taxonomy
V4	Fall	46	0.424	Ciliophora, peritrichia, *Opercularia microdiscum*
	Fall	45	0.425	Stramenopiles, *Diplophrys sp. ATCC 50360*
	Winter	42	0.417	Fungi, *Yarrowia sp. TFM01*
	Winter	40	0.416	RT5iin25, uncultured freshwater eukaryote
	Winter	40	0.415	Ciliophora, phyllopharyngea, uncultured phyllopharyngid ciliate
	Spring	34	0.394	Cercozoa, *Lecythium sp*.
	Spring	33	0.391	Unidentified
	Summer	32	0.396	Alveolata, *Colpodella edax*
	Summer	30	0.392	Fungi, *Cyberlindnera jadinii*
	Summer	30	0.393	Unidentified
	Summer	30	0.395	Cercozoa, *Euglypha rotunda*
V9	Fall	30	0.406	Unidentified
	Fall	30	0.406	Excavata, kinetoplastea, *Parabodo caudatus*
	Winter	31	0.397	Unidentified
	Winter	32	0.395	Unidentified
	Spring	33	0.410	Excavata, *Gyropaigne lefevrei*
	Spring	33	0.402	Amoebozoa, *Entamoeba coli*
	Spring	33	0.401	Ciliophora, peritrichia, *Vorticella microstoma*
	Summer	30	0.405	Unidentified
	Summer	30	0.405	Unidentified
	Summer	32	0.403	Unidentified

### Host population dynamics are reflected in sewage communities

**Fig. 6:**
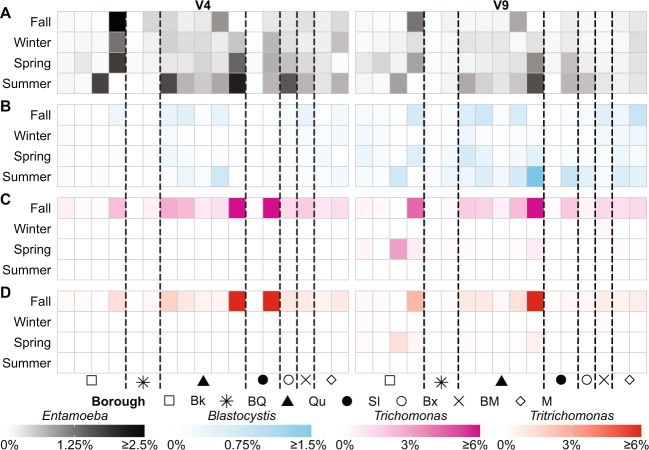
Relative abundances of genera of interest in DEP sewage samples over time from 18S rRNA gene amplicon sequencing. For all heat maps rows indicate collection season, columns represent samples, and each cell shows the average relative abundance from two replicate samples. **a** *Entamoeba* relative abundance. **b** *Blastocystis* relative abundance. **c** *Trichomonas* relative abundance. **d** *Tritrichomonas* relative abundance. In all panels lines separate samples by borough. Abbreviations: Bk = Brooklyn, BQ = Brooklyn/Queens, Qu = Queens, SI = Staten Island, Bx = Bronx, BM = Bronx/Manhattan, M = Manhattan

Human- and animal-associated taxa showed significant variation spatially and temporally across sewage samples. Species of *Entamoeba* occurred in all samples, and *Blastocystis* was found in the majority of samples at low relative abundances (mean < 1%) but occasionally spiking to >1% in several samples (Fig. 6). The V4 and V9 data showed similar patterns, with higher relative abundances observed in the spring and summer compared to fall and winter samples. Higher overall levels of *Entamoeba* were present in the V4 data, while the V9 data had higher relative abundances of *Blastocystis* (Fig. 6a, b), likely due to the detection of *Entamoeba histolytica* and *Entamoeba suis* in the V4 data that were not recovered in the V9 data. Parabasalids (*T. foetus* and *Trichomonas spp*.) were also observed but less frequently, and primarily in fall samples; however, the V9 probe detected a resurgence of these taxa in samples from Brooklyn and Queens in the spring (Fig. 6c, d). We did not detect high relative abundances of other common gut taxa including *Dientamoeba fragilis*, *Cryptosporidium*, and *Giardia* in the DEP sewage samples.

## Discussion

This report is the first comprehensive study of protists sampled from raw sewage collected at several time points over one year from the largest city in the United States, and several insights were gained. First, sewage samples were found to be dominated by free-living protist clades likely introduced due to the combined nature of the NYC sewage system, including ciliates, chrysophytes, cercozoans, and kinetoplastids, members of which are abundant, ecologically significant, and have been isolated from other environments [17, 20, 41, 57]. Protist populations were also highly differentiated by environment, with distinct divides between DEP sewage, private sewage, soil, and aquatic samples. Thus, protists are probably introduced to the sewage community through rainwater and stormwater infiltration, although these may also represent transient taxa that are “passing through” the environment. Human and animal microbiomes represent another source of sewage microbes, but these taxa were present at much lower relative abundances compared to nonhuman taxa, possibly explained by the lower diversity of gut protists in United States populations [58], or unfavorable conditions for proliferation of these microbes in sewer infrastructure.

Second, several taxa such as OTUs representing oligohymenophorean ciliates had the highest relative abundance in NYC sewage regardless of geography or season, consistent with their role as common members of raw sewage [20, 21]. The consistency of these OTUs across samples suggests they may be residents of the sewage infrastructure, and although these taxa are also commonly found in freshwater and soil habitats, they were not detected at high levels in the other environments examined in this study. Previous studies have shown that pipe-based sewage systems maintain natural bacterial communities in biofilms or sediments, and as generalist bacterivores these protist taxa may exist as predators to this community [14, 20, 59–61]. Other potential resident taxa such as the uncharacterized core community may also be unique to the NYC sewer environment. Although they were not present at high relative abundances, these uncharacterized OTUs could represent dormant taxa that grow to abundance under specific environmental conditions not experienced during our sampling period, or have a specialized niche in sewage ecosystem function. Overall, these observations suggest that the primary contributors to the influent of the NYC wastewater treatment plants are nonhuman in origin, some of which may be residents of and unique to NYC or urban sewage infrastructure. This accords with previous studies that estimated 80–90% of bacterial sequences in sewage to be nonfecal in origin [13, 14, 62]. Further studies will be needed to determine if these OTUs are truly unique to sewage systems or present in natural systems at very low relative abundances.

Third, although the same taxonomic clades dominated throughout the year, seasonal patterns were observed, explained by the low relative abundance of the protist core community. Previous studies of sewage bacterial microbiomes have found a higher degree of conservation across sites, with up to 70% of total reads attributed to core OTUs while only 47–60% of the total reads were attributed to core protists in this study [14, 59, 62]. Our results are also consistent with previous studies that showed no or small correlations between bacterial community composition, plant chemical and physical measurements, average inflow, and population size served [13, 14]. A few NYC sewage samples within each season were also very dissimilar to each other, and these protist communities may therefore be sensitive to transient taxa that are “passing through” from other environments. Further sampling with greater short- and long-term longitudinal depth will be required to determine more accurate estimates of what taxa are part of the stable versus transient population.

Fourth, the microbial functional composition of NYC sewage was similar between samples. The most abundant functional categories in sewage were those related to nucleotide metabolism and amino acid metabolism, core metabolic functions that are conserved across microbial communities from a variety of habitats [63]. Our shotgun metagenomic data suggest that sewage microbial function is more consistent than its community structure, as has been reported for the surface of seaweed and the human microbiome [64, 65]. A similar trend was observed in our network analysis, where individual varied between seasons, but the overall network was robust regardless of probe or collection season. Further investigation into these core functions as well as the less abundant noncore functions will be needed to illuminate the interdependencies of this community such as taxa that perform similar or complementary functions, trophic interactions between predators and prey, symbioses, competition, or shared environmental drivers.

Our network analysis, which identified several potential keystone taxa that did not have high relative abundances and were not identified as core OTUs in other analyses, suggests a situation in which essential pathways are conserved across environments but the microbes responsible for them are not, and key functional contributions may be performed by minor or less abundant taxa, which has also been reported in other studies [66]. This further emphasizes the importance of rare microorganisms in sewage, which could serve as a reservoir for genetic or functional diversity and/or a buffer against environmental change [67]. Together our results provide support for the notion of a core metagenome in which the overall population structure, behavior, and function may be more essential to sewage protist communities than species abundance. Additional large-scale studies, including generating transcriptomic and metabolomic data across multiple NYC environments, will deepen our understanding of this complex microbial system and could offer unique new tools for water quality monitoring and wastewater management.

Previous comparisons of sewage bacterial communities demonstrate that sewage accurately reflects the microbial composition of human stool and can capture population level traits that cannot be readily observed by epidemiology studies that sample a limited number of individuals [11–13]. We made similar observations for the protist community present in NYC sewage. Protists are common inhabitants of human and animal microbiomes, and we expected to detect several taxa, including species of *Entamoeba*, *Blastocystis*, *Cryptosporidium*, *Giardia*, and parabasalids, which are typically associated with human and animal guts or feces in NYC sewage samples [18, 58, 68]. We consistently detected common commensal gut taxa of multiple different host species, including *E. coli*, *E. dispar*, and *E. suis*, in all NYC sewage samples. *Blastocystis*, long considered a pathogen and associated with irritable bowel syndrome [69] (although evidence suggests that it may represent a normal component of human and animal flora) [70], was also detected in NYC sewage samples, although less frequently than *Entamoeba*, which may reflect low prevalence in the United States relative to other industrialized populations [71]. Data on the prevalence of commensal *Entamoeba* species in western human populations is not available.

We also detected some taxa of public health importance in NYC sewage. *E. histolytica* (a zoonotic parasite of the digestive tract [72]) was observed in low relative abundances in numerous samples but occasionally spiked to >2% in several samples in the fall, spring, and summer. The New York State Department of Health Communicable Disease Annual Report reported 458 diagnosed cases of amebiasis, caused by *E. histolytica*, in 2014 and 417 in 2015. We also observed parabasalids (*T. foetus* and *Trichomonas sp*.) at high relative abundances in several fall samples. These parabasalids exhibit zoonotic characteristics; however, human (*T. vaginalis*) and other closely related mammalian, e.g., *T. tenax* and avian *Trichomonas* species cannot be differentiated using variable regions of the 18S rRNA gene alone [23]. *T. vaginalis*, although the most common sexually transmitted human parasite, is not a reportable disease, so community health data do not exist for comparison. We did not detect high relative abundances of other gut taxa including *D. fragilis*, *Cryptosporidium*, and *Giardia* in our NYC sewage samples. *D. fragilis* has a variable prevalence worldwide and may be low in New York compared to other areas [73]. Although the 18S rRNA gene primers used here can amplify *Giardia* DNA [23], there are four mismatches between the *Giardia* 18S rRNA gene sequence and the V4 reverse primer and three mismatches to the V9 reverse primer, which may cause low efficiency of *Giardia* DNA amplification from a complex medium like sewage. Finally, the DNA extraction method used in this study (bead beating) may have been insufficient to break open *Cryptosporidium* cysts, which require freeze thaw cycles [74].

The results of this study demonstrate that NYC sewage is a good model system for identifying human and environmental microbes that can be used to track community patterns that are linked to public health. The data also provide a baseline of protist diversity in NYC for future investigations of protists in urban environments. Our study is only a snapshot of protist diversity (living or dead) in raw sewage, and our data are constrained by the limited protist sequence data in public databases, thus establishing a “typical” microbial profile will require further large-scale studies with greater longitudinal depth. Additional, complementary analyses will be needed to determine how much of the measured DNA originates from living protists. Specific assays are needed to confirm taxa of public health importance and their pathogenic potential. In addition, many of these protists can infect both animals and humans, providing no information regarding the source of these microbes. Together with other studies, this work helps to characterize the urban microbiome and provides some understanding of the distribution of zoonotic protists in urban environments.

## Supplementary information

Supplementary Methods

Supplementary Figures

Table S1

Table S2

Table S3

## Data Availability

Raw Illumina sequence data are in NCBI SRA under BioProjects PRJEB23950 (V4), PRJEB26690 (V9), and PRJEB28033 (shotgun data). Documentation of QIIME and R workflows and some data outputs can be found on Github (https://github.com/jmmaritz/NYC-Sewage).
