# Supplementary material for: Patterns of protist diversity associated with raw sewage in New York City
Source: ISME J. 2019 Jul 9;13(11):2750–63. doi: 10.1038/s41396-019-0467-z (PMC6794324; doi:10.1038/s41396-019-0467-z)
Supplement: Supplementary file 1 — Supplementary Methods [file 41396_2019_467_MOESM1_ESM.docx]

**Supplementary Methods**

**Patterns of protist diversity associated with raw sewage in New York City**

Julia M. Maritz^1^, Theresa A. Ten Eyck^1^, S. Elizabeth Alter^2^, and Jane M. Carlton^1^

^1^Center for Genomics and Systems Biology, Department of Biology, New York University, New York, NY, USA; ^2^Department of Biology, York College, City University of New York, New York, NY, USA.

Correspondence: Jane Carlton, [jane.carlton@nyu.edu](mailto:jane.carlton@nyu.edu)

**Sample collection, processing and DNA extraction**

Raw sewage samples were collected from the main influent pipe by DEP personnel at each plant according to standard collection procedures for influent chemical/physical measurements. Composite surface soil samples were collected from multiple locations per site using a trowel and placed into a plastic bucket and homogenized using a 4 mm sieve prior to DNA extraction. Sediment was collected from the Gowanus Canal in Brooklyn at three different locations and depths (-2.5 ft, -7.5 ft, -8.5ft) and combined into one composite sample prior to DNA extraction.

**18S rRNA gene library preparation**

The V4 region was amplified with Illumina primer constructs containing the TAReuk454FWD1 and TAReukREV3 primers. Library synthesis and amplification using 5 ul of input DNA were performed in triplicate using Phusion High-Fidelity PCR Master Mix (Thermo Fischer Scientific, catalog #F-531S), a 20 uL reaction volume, and a two-step PCR amplification strategy as described in (Stoeck *et al.*, 2010): 98°C for 30 seconds, 10 cycles of 98°C for 10 seconds, 53°C for 30 seconds, 72°C for 30 seconds; and then 25 cycles of 98°C for 10 seconds, 48°C for 30 seconds, 72°C for 30 seconds, and ending at 72°C for 10 minutes.

The V9 fragment of the 18S rRNA gene was amplified using Illumina primer constructs containing the universal primers 1391f-EukBr (Amaral-Zettler, McCliment, Ducklow, & Huse, 2009). For the V9 region, DNA from sewage samples was amplified with the addition of the mammal blocking primer, while DNA from the water, soil and sediment samples was amplified without it (no blocking primer is available for the V4 region). Library synthesis and amplification using 5 ul of input DNA was done in triplicate following the Earth Microbiome protocol.

Extraction kit blanks were PCR amplified for both regions, and no-template (water blank) PCR controls were performed for all PCR reactions. No bands were visible on agarose gels after amplification and no DNA was detected using the Qubit dsDNA HS Assay kit (Thermo Fischer Scientific, catalog #Q32851), thus these control samples were excluded from downstream Illumina sequencing.

After amplification, triplicate PCR reactions were pooled and purified with a 1.8X ratio of AMPure XP beads (Beckman Coulter, catalog #A63880) on the Bravo NGS Workstation (Agilent Technologies). The size distribution of purified libraries was determined using the TapeStation 2200 (Agilent Technologies), and quantified via Quant-iT dsDNA Assay Kit (Thermo Fischer Scientific, catalog #Q33120). Quantified libraries were individually normalized to 4 nM and equal volumes of each 4 nM dilution were pooled for sequencing.

MiSeq preparation and sequencing was performed based on the manufacturer’s and Earth Microbiome protocols (Caporaso *et al.*, 2012) using the following parameters. Pools for the V4 region were sequenced at a final concentration of 12 pM with a 10% PhiX control spike-in using an Illumina MiSeq 500 cycle V3 kit and 2x300 run configuration. Pools for the V9 region were sequenced at a final concentration of 10 pM with a 6% PhiX control spike-in using an Illumina MiSeq 300 cycle V2 reagent kit with a 2x100 run configuration.

**Shotgun metagenomic library preparation**

For 15 of the 17 November 2014 samples, DNA from the biological replicates was pooled to increase the biomass input for library construction. In one case, due to very low biomass, DNA from the four biological replicates representing one plant (regions 4 and 9, **Fig. 1**) was pooled, resulting in a total of 16 samples. Pooled input DNA was fragmented to ~500 bp using a Covaris sonicator and libraries were constructed using the KAPA LTP Library Preparation Kit (KAPA Biosystems, catalog #KK8232) according to the manufacturer’s instructions. To improve the recovery of DNA fragments and proportion of input DNA converted to adapter-ligated molecules, the AMPure XP bead-based cleanups following the end-repair and A-tailing reactions were increased to a 2X ratio and the ligation reaction time was increased to 30 minutes. Libraries were then size selected using a 0.4X ratio followed by a 0.15X ratio of AMPure XP beads to retain adapter-ligated fragments between 400 bp and 800 bp in length and PCR amplified for 5-10 cycles based on the input concentration of fragmented DNA. The size distribution of purified libraries was determined using the 2200 TapeStation and quantified via the Qubit dsDNA HS Assay kit.

**18S rRNA gene amplicon sequence data analysis**

Alpha diversity was calculated on OTU tables rarefied to 50,000 sequences per sample for the V4 region and 90,000 sequences per sample for the V9 region using the Shannon diversity index within the QIIME pipeline. Beta diversity and ordinations were calculated by Non-Metric Multidimensional Scaling (NMDS) using the Bray-Curtis dissimilarity in R with the packages vegan(v2.4-3) (Oksanen *et al.*, 2017) and Phyloseq(v1.20) (McMurdie & Holmes, 2013). The association of community composition with metadata factors was assessed with non-parametric multivariate analysis of variance (adonis) tests in R with 10,000 permutations. All other downstream analyses were conducted on sum-normalized OTU tables. Univariate tests for differentially abundant taxa with respect to environment was performed using LEfSe (Segata *et al.*, 2011) with an alpha value of 0.05, all-against-all comparison, and a LDA cutoff of 2.0. GraPhlAn (Asnicar, Weingart, Tickle, Huttenhower, & Segata, 2015) was used for visualization of the LEfSe results and phylogenetic relationships.

**Network analysis**

Network analysis was performed in R using the SpiecEasi (v0.1.2) (Kurtz *et al.*, 2015) package with the neighborhood selection (mb) mode, individual on per season OTU tables filtered to remove OTUs present in less than one third (eleven) samples. Model selection was performed via StARs using nlambda=20 and 50 repetitions. Network characterization (similarity and robustness) was evaluated using different topological properties: number of nodes (OTUs), number of edges (predicted interactions), number of connected components, mean distance, diameter, degree, density, closeness centrality and centralization.

- The number of **connected components** provides the number of clusters (groups of connected nodes) in a network.
- **Distance** calculates the shortest path length between any two nodes in the network as the path with the fewest edges between them. Mean distance represents the mean number of edges between any two nodes in the network.
- **Diameter** is the longest path length, in number of edges, between the furthest two nodes in the network.
- **Degree** counts the number of edges that are connected to each node in the network.
- **Density** is equal to the proportion of edges present in the network out of all possible edges and is a measure of network connectivity based on the individual components.
- **Closeness centrality** measures how central a node is to the network as the average number of shortest paths between that node and all other nodes in the network. Closeness centrality was normalized by the number of possible edges per node to allow for comparison between nodes from networks of different sizes.
- **Centralization** is a method for calculating network wide centrality based on node level properties.

**References**

Amaral-Zettler, L. A., McCliment, E. A., Ducklow, H. W., & Huse, S. M. (2009). A method for studying protistan diversity using massively parallel sequencing of V9 hypervariable regions of small-subunit ribosomal RNA genes. *PLoS One, 4*(7), e6372. doi:10.1371/journal.pone.0006372

Asnicar, F., Weingart, G., Tickle, T. L., Huttenhower, C., & Segata, N. (2015). Compact graphical representation of phylogenetic data and metadata with GraPhlAn. *PeerJ, 3*, e1029. doi:10.7717/peerj.1029

Caporaso, J. G., Lauber, C. L., Walters, W. A., Berg-Lyons, D., Huntley, J., Fierer, N., . . . Knight, R. (2012). Ultra-high-throughput microbial community analysis on the Illumina HiSeq and MiSeq platforms. *ISME J, 6*(8), 1621-1624. doi:10.1038/ismej.2012.8

Kurtz, Z. D., Muller, C. L., Miraldi, E. R., Littman, D. R., Blaser, M. J., & Bonneau, R. A. (2015). Sparse and compositionally robust inference of microbial ecological networks. *PLoS Comput Biol, 11*(5), e1004226. doi:10.1371/journal.pcbi.1004226

McMurdie, P. J., & Holmes, S. (2013). phyloseq: an R package for reproducible interactive analysis and graphics of microbiome census data. *PLoS One, 8*(4), e61217. doi:10.1371/journal.pone.0061217

Oksanen, J., Blanchet, F. G., Friendly, M., Kindt, R., Legendre, P., McGlinn, D., . . . Wagner, H. (2017). *vegan: Community Ecology Package. Ordination methods, diversity analysis and other functions for community and vegetation ecologists. Version 2.4-3. URL* [*https://CRAN.R-project.org/package=vegan*](https://CRAN.R-project.org/package=vegan).

Segata, N., Izard, J., Waldron, L., Gevers, D., Miropolsky, L., Garrett, W. S., & Huttenhower, C. (2011). Metagenomic biomarker discovery and explanation. *Genome Biol, 12*(6), R60. doi:10.1186/gb-2011-12-6-r60

Stoeck, T., Bass, D., Nebel, M., Christen, R., Jones, M. D., Breiner, H. W., & Richards, T. A. (2010). Multiple marker parallel tag environmental DNA sequencing reveals a highly complex eukaryotic community in marine anoxic water. *Mol Ecol, 19 Suppl 1*, 21-31. doi:10.1111/j.1365-294X.2009.04480.x
