# Supplementary material for: Patterns of protist diversity associated with raw sewage in New York City
Source: ISME J. 2019 Jul 9;13(11):2750–63. doi: 10.1038/s41396-019-0467-z (PMC6794324; doi:10.1038/s41396-019-0467-z)
Supplement: Supplementary file 2 — Supplementary Figures [file 41396_2019_467_MOESM2_ESM.pdf]

Supplementary Figures

Patterns of protist diversity associated with raw sewage in New York City

Julia M. Maritz[1], Theresa A. Ten Eyck[1], S. Elizabeth Alter[2], and Jane M. Carlton[1]

[1]Center for Genomics and Systems Biology, Department of Biology, New York University, New York, NY, USA;

[2]Department of Biology, York College, City University of New York, New York, NY, USA.

Correspondence: Jane Carlton, jane.carlton@nyu.edu

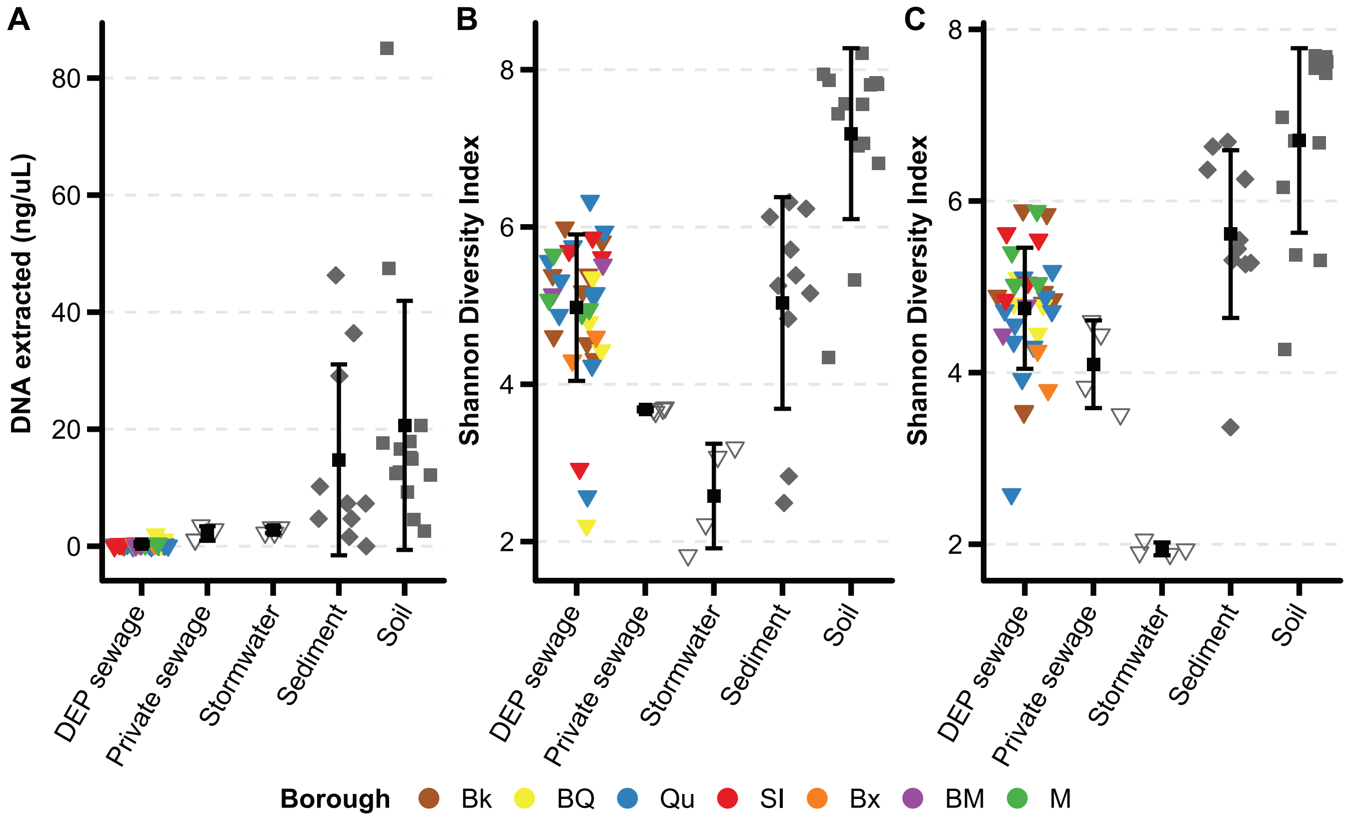

Fig. S1 Biomass and alpha diversity analyses of 18S rRNA gene amplicon sequence data for DEP sewage, private sewage, water, sediment, and soil samples. A) Biomass of the different environments plotted as DNA yield (ng/uL). B) Alpha diversity of the different environments as measured by the Shannon Diversity index for the V4 region. Data was rarefied to 50,000 sequences per sample. C) Alpha diversity of the different environments as measured by the Shannon Diversity index for the V9 region. Data was rarefied to 90,000 sequences per sample. Colors represent the borough from which each DEP sample was collected. Triangle = private building; diamond = sediment; square = soil. Abbreviations: Bk = Brooklyn, BQ = Brooklyn/Queens, Qu = Queens, SI = Staten Island, Bx = Bronx, BM = Bronx/Manhattan, M = Manhattan.

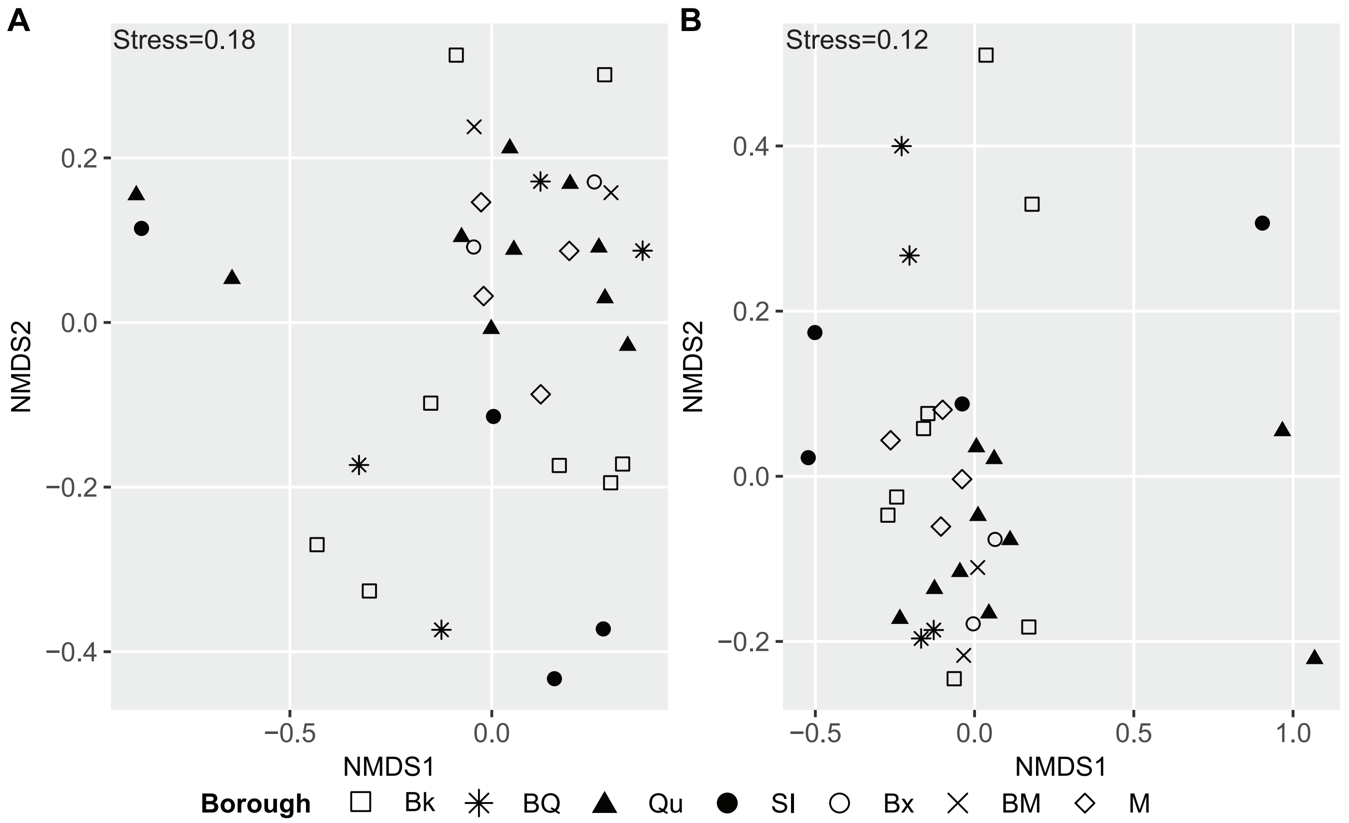

Fig. S2 NMDS of Bray-Curtis dissimilarity for DEP sewage samples. A) V4 region and B) V9 region. In both panels shapes represent the borough from which each DEP sample was collected. Abbreviations: Bk = Brooklyn, BQ = Brooklyn/Queens, Qu = Queens, SI = Staten Island, Bx = Bronx, BM = Bronx/Manhattan, M = Manhattan.

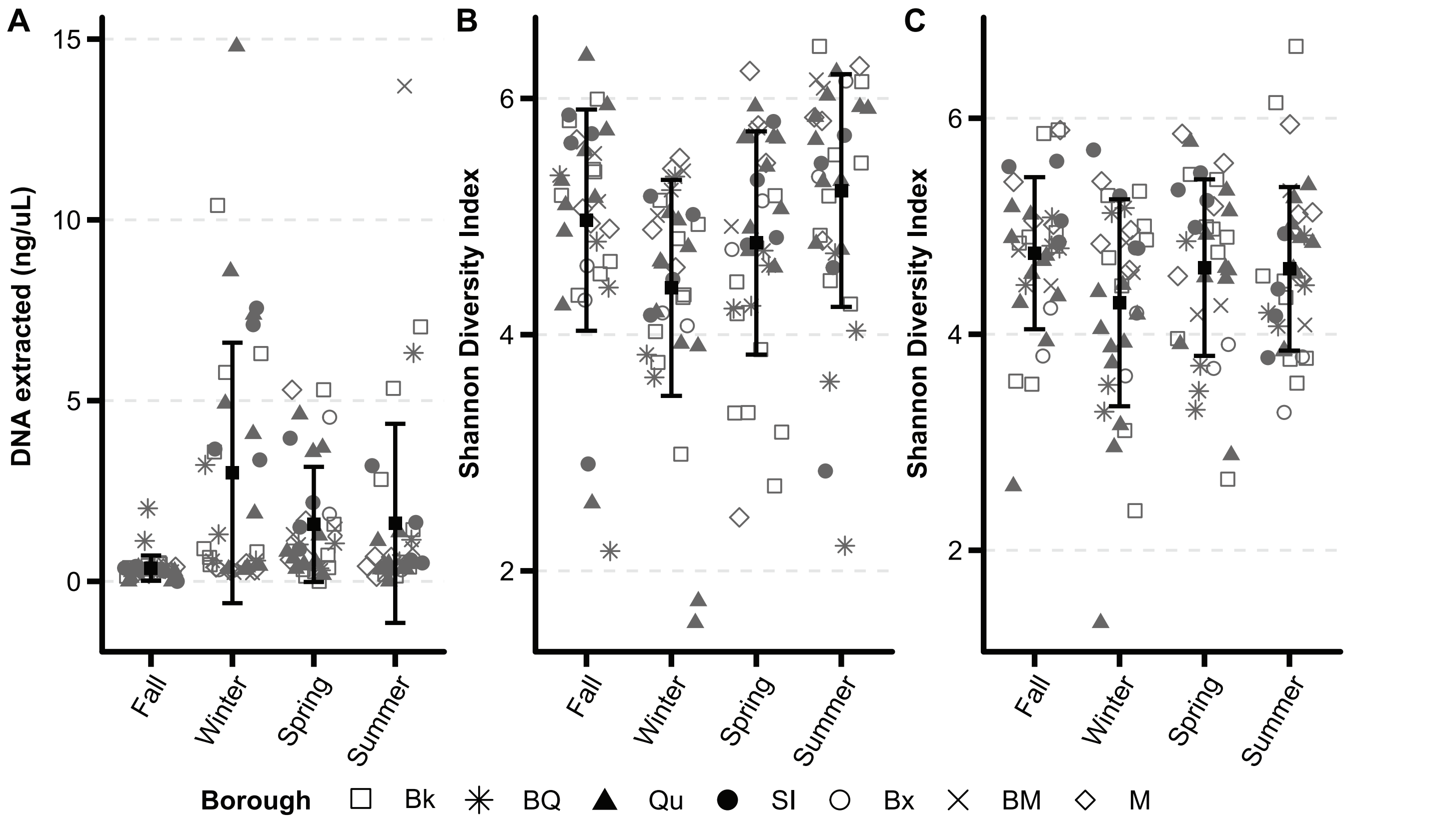

Fig. S3 Biomass and alpha diversity for DEP sewage samples by collection season from 18S rRNA gene amplicon sequencing. A) Biomass per season. DNA yield is plotted in nanograms per microliter. B) Alpha diversity per season as measured by the Shannon Diversity index for the V4 region. Data was rarefied to 50,000 sequences per sample. C) Alpha diversity per seasons measured by the Shannon Diversity index for the V9 region. Data was rarefied to 90,000 sequences per sample. In all panels shapes represent the borough where DEP sample was collected. Abbreviations: Bk = Brooklyn, BQ = Brooklyn/Queens, Qu = Queens, SI = Staten Island, Bx = Bronx, BM = Bronx/Manhattan, M = Manhattan.

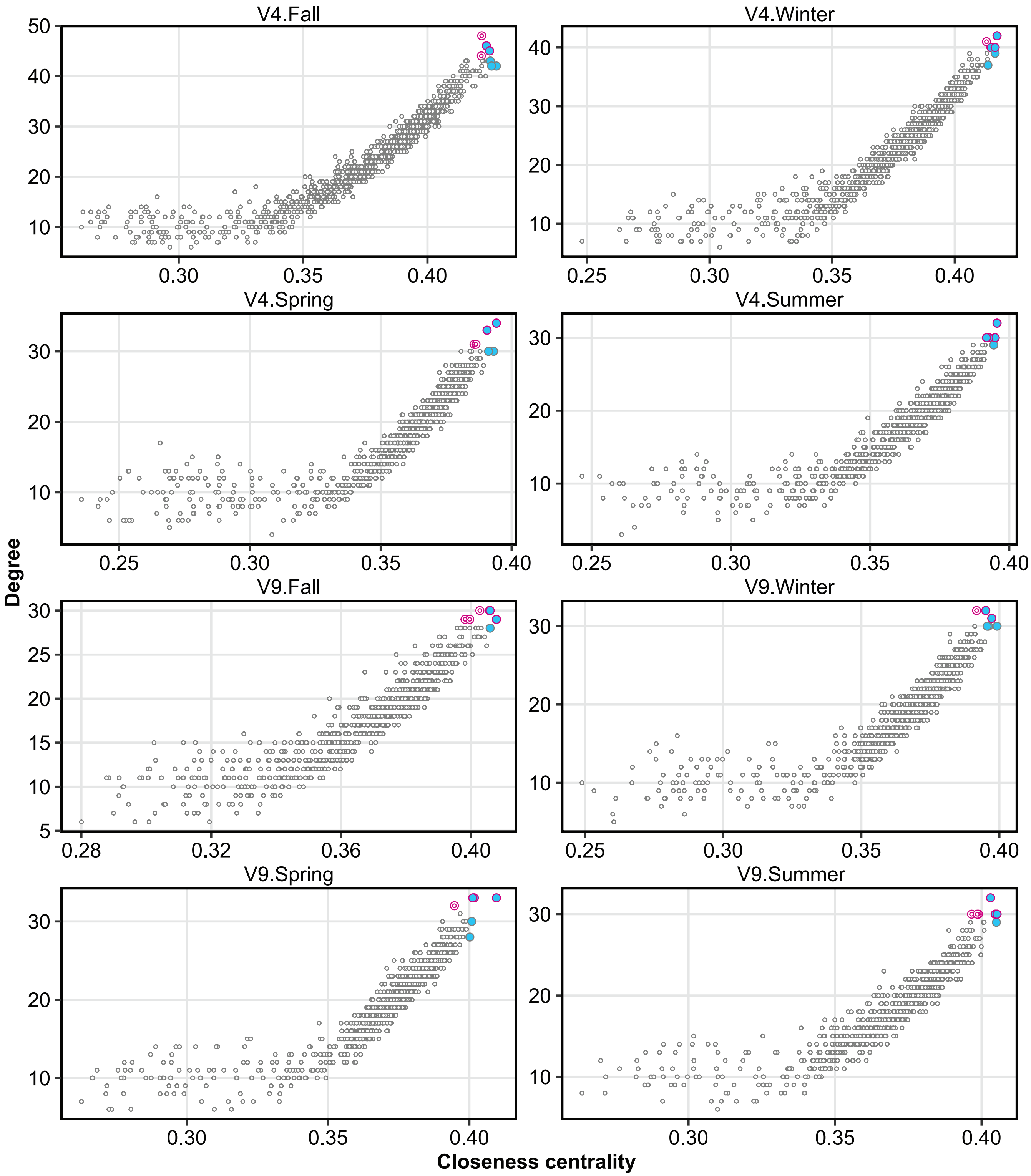

Fig. S4 Closeness centrality vs. degree for DEP networks by season from 18S rRNA gene amplicon sequencing. Plots show the closeness centrality and degree for each node in the network. Nodes with the highest closeness centrality are blue, those with the highest degree are outlined in pink. Those that overlap have both. The top OTUs by closeness were not always consistent with degree. Those that overlap are potential keystone species.
